# *Pseudomonas aeruginosa* LptE is crucial for LptD assembly, cell envelope integrity, antibiotic resistance and virulence

**DOI:** 10.1080/21505594.2018.1537730

**Published:** 2018-11-04

**Authors:** Alessandra Lo Sciuto, Alessandra M. Martorana, Regina Fernández-Piñar, Carmine Mancone, Alessandra Polissi, Francesco Imperi

**Affiliations:** a Department of Biology and Biotechnology Charles Darwin, Sapienza University of Rome, Laboratory affiliated to Istituto Pasteur Italia – Fondazione Cenci Bolognetti, Rome, Italy; b Department of Pharmacological and Biomolecular Sciences, University of Milan, Milan, Italy; c Department of Cellular Biotechnologies and Haematology, Sapienza University of Rome, Rome, Italy

**Keywords:** *Galleria mellonella*, infection, lipid A, LPS transport, LptD, LptH, outer membrane, resistance, virulence

## Abstract

Lipopolysaccharide (LPS) is an essential structural component of the outer membrane (OM) of most Gram-negative bacteria. In the model organism *Escherichia coli*, LPS transport to the OM requires seven essential proteins (LptABCDEFG) that form a continuous bridge across the cell envelope. In *Pseudomonas aeruginosa* the recently-demonstrated essentiality of LptD and LptH, the *P. aeruginosa* LptA homologue, confirmed the crucial role of the Lpt system and, thus, of LPS in OM biogenesis in this species. Surprisingly, independent high-throughput transposon mutagenesis studies identified viable *P. aeruginosa* insertion mutants in the *lptE* gene, suggesting that it might be dispensable for bacterial growth. To test this hypothesis, we generated an *lptE* conditional mutant in *P. aeruginosa* PAO1. LptE depletion only slightly impairs *P. aeruginosa* growth *in vitro*. Conversely, LptE is important for cell envelope stability, antibiotic resistance and virulence in an insect model. Interestingly, the maturation and OM localization of LPS is only marginally affected in LptE-depleted cells, while the levels of the OM component LptD are strongly reduced. This suggests that *P. aeruginosa* LptE might not be directly involved in LPS transport, although it is clearly essential for the maturation and/or stability of LptD. While poor functionality of LptD caused by LptE depletion is somehow tolerated by *P. aeruginosa*, this has a high cost in terms of cell integrity, drug resistance and virulence, highlighting LptE function(s) as an interesting target to weaken *P. aeruginosa* defenses and reduce its infectivity.

## Introduction

Gram-negative bacteria are characterized by a cell envelope consisting of two concentric membranes, the inner (IM) and outer membrane (OM), which show distinct composition, and structural and functional properties [1]. The two lipid bilayers delimit an aqueous compartment, the periplasm, containing a thin layer of peptidoglycan [2]. While the IM is a typical phospholipids bilayer, the OM is an asymmetric membrane composed of glycerophospholipids in the inner leaflet, and lipopolysaccharide (LPS) in the outer leaflet [1]. LPS is a negatively charged glycolipid; in the presence of divalent cations LPS molecules form a tightly packed layer at the outer leaflet thus reducing OM fluidity and permeability, which is selectively controlled by dedicated OM proteins [3]. Perturbation of LPS organization at the cell surface by defects in LPS biogenesis and/or impaired assembly of OM components result in loss of membrane integrity due to i) migration of phospholipids from the inner to the outer leaflet of the OM causing locally symmetric bilayer rafts, and ii) generation of transient imperfections or “cracks” which can allow the entry of both lipophilic and hydrophilic compounds [4,5]. The asymmetry of the OM is generated by the Lpt (Lipopolysaccharide transport) system, a molecular machine composed of seven essential proteins in *Escherichia coli* (LptABCDEFG) that ferries LPS from the periplasmic side of the IM, across the periplasm to the cell surface [6]. The Lpt proteins assemble to form a multiprotein complex that spans the entire cell envelope [7]. This is organized in two sub-assemblies, LptB_2_CFG and LptDE, located at the IM and at the OM, respectively [8–11], which are connected by the periplasmic protein LptA [12–14]. At the IM the LptB_2_FG ATP-binding cassette (ABC) transporter, associated to the bitopic protein LptC, powers the LPS export to the cell surface [15,16]. At the OM, the β-barrel protein LptD and the lipoprotein LptE constitute the OM translocon, characterized by a peculiar plug-and-barrel architecture [17–19]. LPS extracted from the IM by the LptB_2_FG ABC transporter is sequentially transferred to LptC and then to LptA in an energy-dependent process [15,16]. Lastly, LPS is thought to be delivered to the LptDE OM translocon for its final assembly at the outer leaflet [20]. It is well established that LptE plays an essential role in the assembly of functional LptD [20–24]. However, more recently LptE has been shown to play a role also in the LPS export process in *E. coli*, as witnessed by its ability to bind LPS [20] and disaggregate it *in vitro* [25]. While the LPS transport machinery has been extensively characterized in *E. coli*, little is known about its functional conservation in other Gram-negative bacteria. At present, comprehensive investigation of Lpt components has only been carried out in *Neisseria meningitidis*, an LPS-producing Gram-negative bacterium for which LPS is however not essential for viability and OM biogenesis [26–28]. Systematic analysis of LPS transport genes in *N. meningitidis* revealed that, despite being dispensable for cell viability in line with the non-essential role of LPS in this species, LPS transport proteins are all essential for LPS transfer to the OM, with the only exception of LptE [24,29,30]. Indeed, deletion of the *lptE* gene in *N. meningitidis* does not impair transport of LPS to the cell surface, although it affects total levels of LptD, suggesting a conserved chaperone-like role for LptE in LptD biogenesis [24]. Evidences on the role of the Lpt machinery have also been accumulated in the Gram-negative bacterium *Pseudomonas aeruginosa*, an opportunistic pathogen responsible for severe infections in immunocompromised and cystic fibrosis patients which are poorly responsive to antibiotic therapies [31]. The first experimental proof of the relevance of the *P. aeruginosa* Lpt complex came from the serendipitous finding that LptD was the molecular target of a peptidomimetic antibiotic with potent anti-*P. aeruginosa* activity [32], and from subsequent confirmation of the essentiality of LptD in this species by conditional mutagenesis [33]. More recently, a reverse-genetic screening for uncharacterized essential periplasmic proteins revealed that LptH, the *P. aeruginosa* homologue of *E. coli* LptA [34], is crucial for *P. aeruginosa* growth, cell envelope biogenesis and pathogenicity in different animal models [35]. While these works clearly confirmed the importance of the Lpt machinery, in line with the essentiality of LPS biosynthesis genes and, thus, of LPS *in P. aeruginosa* [36,37], the role of other Lpt components in LPS transport remains to be determined. Interestingly, although previous projects aimed at generating saturating libraries of sequence-defined transposon insertion mutants proposed *lptE* as a putative essential gene in this bacterium [38,39], two recent transposon-sequencing (Tn-seq) studies detected viable *P. aeruginosa lptE* transposon insertion mutants under certain growth conditions [40,41]. This finding suggests that the *lptE* gene might be dispensable for *P. aeruginosa* growth. However, considering that Tn-seq has some limitations, including the inability to distinguish mutants whose phenotypes are “complemented” by other bacteria in the transposon-mutant pool [40], confirmatory experiments with individual mutants are crucial to verify Tn-seq findings. In this work, we employ a conditional mutagenesis approach to investigate the effect of LptE depletion on the physiology of *P. aeruginosa*. LptE-depleted *P. aeruginosa* PAO1 cells are only slightly impaired in *in vitro* growth, while they are strongly defective in the ability to cause infection in an animal model. LPS transport in *P. aeruginosa* is only marginally affected by LptE depletion, although LptE is confirmed to play an important role as LptD chaperone. Notably, detergent and antibiotics sensitivity is drastically increased in LptE-depleted cells, likely because of improperly folded and/or un-plugged LptD channels in the OM.

## Materials and methods

### Bacterial strains and growth conditions

Bacterial strains and plasmids used in this study are listed in Table S1. Bacteria were cultured in Lysogeny Broth, Lennox formulation (LB; Acumedia) for genetic manipulation, while growth assays were performed in Mueller-Hinton broth (MH; Difco), LB or M9 minimal medium supplemented with 20 mM succinate as carbon source and 50 µM FeCl_3_ [42]. When specified, growth media were supplemented with L-arabinose, or IPTG at the indicated concentrations. Growth assays in liquid cultures were performed in 96-well microtiter plates (200 µL of medium in each well) or in flasks at 37°C and vigorous shaking (200 rpm). Growth was measured as the optical density at 600 nm (OD_600_) of bacterial cultures in a Victor plate reader (Wallac) for microtiter plates or of appropriate dilutions in sterile growth medium in a spectrophotometer for flask cultures. Growth assays on solid media were performed by spotting 5 µL of serial ten-fold dilutions from bacterial suspensions in saline normalized to an OD_600_ = 1, from late-exponential cultures grown in the presence or in the absence of 0.5% (w/v) arabinose on media solidified with 1.5% (w/v) agar. When required, antibiotics were added at the following concentration for *E. coli* (the concentration used for *P. aeruginosa* is shown between brackets): ampicillin 100 µg/mL, nalidixic acid 20 µg/mL, chloramphenicol 30 µg/mL (350 µg/mL), tetracycline 12 µg/mL (50–100 µg/mL).

### Generation of plasmids and recombinant strains

Recombinant DNA procedures have been described elsewhere [42]. All DNA fragments for cloning were amplified by PCR with Pfu (Promega) or Q5 Hot Start High-Fidelity (New England Biolabs) DNA Polymerases using genomic DNA of *P. aeruginosa* PAO1 as the template. Primers and restriction enzymes used for cloning are described in Table S2. All constructs were verified by DNA sequencing. The LptE expressing construct pME*lptE* was generated by cloning the *lptE* coding sequence into the IPTG-inducible shuttle vector pME6032 [43], and introduced in *P. aeruginosa* by transformation using chemically competent cells. The integration-proficient construct mini-CTX1-*araC*P_BAD_*lptE* was generated by replacing the *tolB* gene in mini-CTX1-*araC*P_BAD_*tolB* [44] with the coding sequence of the *lptE* gene. In this construct, the *lptE* coding sequence is cloned downstream of an arabinose-dependent regulatory element *araC*-P_BAD_ optimized for *P. aeruginosa* by modification of the ribosome-binding site [36]. The deletion mutagenesis construct pDM4Δ*lptE* was obtained by directionally cloning two DNA regions upstream and downstream of the *lptE* coding sequence into pBluescript II KS+ (Stratagene), and then subcloning the DNA fragment encompassing the *lptE* upstream and downstream regions into the suicide vector pDM4 [45]. The *lptE* conditional mutant was generated using a recently-described strategy [44]. Briefly, the *lptE* coding sequence under the control of modified arabinose-dependent regulatory element *araC*-P_BAD_ (see above) was integrated into the *attB* neutral site of the *P. aeruginosa* chromosome, and excision of the mini-CTX1 plasmid backbone from the *attB* neutral site was achieved by Flp-mediated recombination as described [46]. In-frame deletion of the endogenous copy of *lptE* was obtained using the *sacB*-based suicide construct pDM4Δ*lptE* as previously described [44] under permissive condition (*i.e*. growth in the presence of 0.5% arabinose). The gene replacement construct pDM4*lptD-6his* was generated by individually cloning two 500-bp DNA regions encompassing either the 3ʹ region of the *lptD* coding sequence (upstream region) or the 5ʹ region of the adjacent gene *surA* (downstream region) into pBluescript II KS+, and then subcloning the upstream and downstream regions into the suicide vector pDM4 by triple ligation. Note that the reverse primer used to amplify the upstream region (*lptD*-6his_UP_RV) contains an additional sequence encoding the 6His tag (Table S2), and that the resulting gene replacement construct pDM4*lptD-6his* includes a 64-bp duplication of the *lptD* 3ʹ end. This was necessary to preserve the ribosome-binding site and the coding sequence of the adjacent gene *surA*, which partially overlaps with *lptD* in the *P. aeruginosa* PAO1 genome (Fig. S1). PAO1 and the *lptE* conditional mutant expressing the recombinant protein LptD-6His (PAO1 *lptD-6his* and *lptE lptD-6his*, respectively; Table S1) were generated by insertion of the *sacB*-based suicide construct pDM4*lptD-6his* followed by sucrose-mediated selection of second recombination events [45]. Clones carrying the *6his-tag* insertion were screened by PCR and then verified by DNA sequencing.

### Detergent sensitivity assay

Sensitivity to the lytic effect of sodium dodecyl sulphate (SDS) was assessed by determining the turbidity (OD_600_) of bacterial cell suspensions in saline after 5-min incubation at room temperature in the presence of increasing concentrations of SDS (0–5%, w/v) [44].

### Antibiotics sensitivity assay

Resistance to the growth inhibitory activity of several antibiotics was assessed by the Kirby-Bauer disc diffusion test. Bacterial cell suspensions in saline were normalized at 0.5 McFarland Standard and swabbed onto MH agar plates supplemented or not with arabinose at the indicated concentration, using disks containing streptomycin (10 µg), ciprofloxacin (5 µg), imipenem (10 µg), colistin (10 µg), novobiocin (30 µg), erythromycin (15 µg), rifampicin (5 µg) and tobramycin (10 µg) (Becton Dickinson). Growth inhibition halo diameters were measured after 20 h of growth at 37°C.

### Fluorescence microscopy

Microscopy images were obtained with a Zeiss Axiovert 200M microscope through a 63 × 1.45 oil objective coupled to a AxioCam Mrm device 290 camera (Zeiss). PAO1, *lptH* and *lptE* conditional mutant cells grown in MH in the absence and/or presence of arabinose were collected from a total amount corresponding to an OD_600_ of 4, and a 1:10 ratio of fixation solution [fixation solution: formaldehyde 37% (v/v) – glutaraldehyde 25% (v/v) in PBS] was added. Cells were incubated for 30 min at 37°C with shaking, washed with PBS and resuspended in 500 µL of PBS. 5 µL of the cell suspensions were spotted onto an agarose-coated glass slide [1% (w/v) agarose], and the samples were covered with a glass coverslip. To stain cell membranes, FM5–95 (ThermoFisher) was added to the agarose solution to a final concentration of 2 µg/mL.

### Cell fractionation and membrane isolation

For cell fractionation and membrane isolation *P. aeruginosa* strains were grown in MH, as described above. The IM and OM were separated by sucrose gradient ultracentrifugation according to published procedures [7,12] with the following modiﬁcations. A total amount of 200 OD_600_ of cells were resuspended in 5 mL of 10 mM Tris/HCl buffer (pH 7.8) containing 25% (w/v) sucrose, 50 µg/mL DNAse, 1 mM PMSF, 100 µg/mL lysozyme. The resuspended cells were lysed by a single passage through cell disruptor (Constant system) at 16,000 psi. The cell lysate was clarified by centrifugation at 3,000 × *g* for 20 min. Cell membranes were collected by centrifuging the supernatant for 1 h at 37,000 rpm in a Sorvall Discovery 90SE with T-890 rotor. Total membranes were resuspended in 1 mL of 10 mM Tris/HCl buffer (pH 7.8) containing 25% (w/v) sucrose and 800 µL were layered on top of the sucrose gradient as described [7]. Fractions (400 µL) were collected from the top of the gradient. The distribution proﬁles of LPS and of XcpY and OprI proteins across the gradient were estimated by western blot as described below. The analysis of LptD levels in LptE-depleted cells was performed on membranes isolated from a total of 20 OD_600_ according to a published protocol [47]. Collected cells were resuspended in 50 mM Tris-HCl, 5 mM EDTA (pH 8.0) containing 1 mM PMSF. After disintegration in cell disruptor (Constant system, 1 passage at 16,000 psi), unbroken cells were removed by centrifugation (3,000 × *g*, 15 min, 4°C). Membrane samples were collected by ultracentrifugation (37,000 in a Sorvall Discovery 90SE with T-890 rotor, 1 h, 4°C) and dissolved in 2 mM Tris-HCl (pH 7.6).

### Lipid A extraction and analysis

Lipid A was extracted from bacterial cell pellets using the ammonium hydroxide-isobutyric acid-based procedure as previously described [48], with few modifications. Briefly, 2 mL of early stationary phase cultures were pelleted and resuspended in 400 μL of 70% (v) isobutyric acid and 1 M ammonium hydroxide (5:3). Samples were incubated for 1 hour at 100ºC and centrifuged at 2,000 x g for 15 min. Supernatants were added to 400 μL of endotoxin-free water, frozen at −80°C, and lyophilized for 4–5 h in a centrifugal vacuum concentrator. The resultant pellet was washed with 1 mL methanol, and the lipid A was extracted using 100 μL of chloroform, methanol, and water (3:1:0.25). After centrifugation at 2,000 x g for 15 min, 2 μL of the supernatants were mixed with 2 μL of 10 mg/mL norharmane matrix in chloroform:methanol (2:1), and 0.5 μL of the mixtures were spotted on a matrix-assisted laser desorption-time of flight (MALDI-TOF) plate (5800 MALDI TOF/TOF Analyzer, Sciex, Ontario, Canada). Samples were analyzed in the negative-ion mode with reflectron mode. Calibration was performed using default calibration originated by five standard spots. Spectral data were analyzed with the 4000 Series Explorer software Version 4.1.0 (Sciex, Ontario, Canada), and used to estimate lipid A forms based on their predicted structures and molecular weights.

### 3-Deoxy-d-manno-oct-2-ulosonic acid (KDO) assay

LPS was extracted from a total of 7 OD_600_ of cells. Cells were collected by centrifugation, the cell pellets were washed twice with 2 mL of sterile phosphate-buffered saline (PBS), resuspended in 500 µL of Hitchcock and Brown lysis buffer [2% (w/v) SDS, 10% (v/v) glycerol and 1 M Tris-HCl, pH 6.8], and heated at 100°C for 30 min. Then, samples were cooled to 20°C and incubated at 56°C for 3 h with 30 µg/mL proteinase K, and proteinase K was inactivated by heating at 95°C for 30 min [49]. The isolated crude LPS was quantified by determining the amount of KDO as previously described [50], using standard curves generated from purified KDO, and normalized to mg of total proteins.

### Western blot analyses

**Figure 1.**
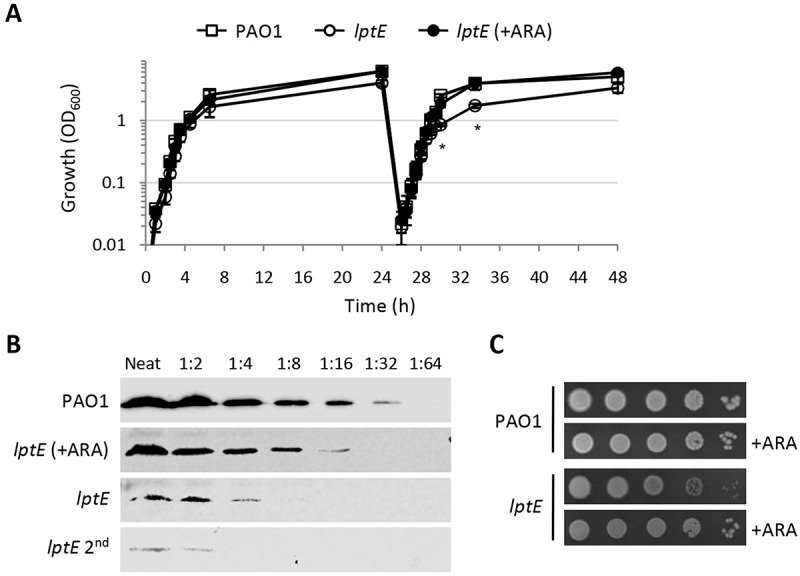
Growth curves, plating efficiency and LptE levels of the wild type strain *P. aeruginosa* PAO1 and the *lptE* conditional mutant. (a) Stationary phase PAO1 and *lptE* conditional mutant (*lptE*) cells grown in MH with 0.5% arabinose (ARA) were diluted 1:1000 in flasks containing MH with or without 0.5% ARA (first refresh). After 24 h of growth, PAO1 and *lptE* cells grown in the absence of ARA were sub-cultured (dilution 1:200) in flasks in fresh medium containing or not 0.5% ARA (second refresh). As the growth curves of PAO1 with or without ARA were basically superimposable, for clarity only the curve in the absence of ARA is shown. Values are the mean (±SD) of three independent experiments. Asterisks indicate the time-points at which the growth of *lptE* without ARA was significantly different from that of other samples (*P *< 0.05, Kruskal-Wallis) (b) Western blot analysis to quantify LptE levels in whole cell lysates (20 µg of proteins; neat), or serial 1:2 dilutions as indicated, from PAO1 or *lptE* conditional mutant cells grown for 14 h in MH with or without ARA for one or two passages (*lptE* 2^nd^). Filters were developed with Cyanagen Westar ηC Ultra 2.0 reagent and visualized in a Odissey system (Li-Cor) system. (c) Colony growth of PAO1 and *lptE* on MH agar plates supplemented or not with ARA. Exponential phase cultures in MH with 0.5% ARA were normalized to OD_600_ = 1 in saline, and 5 μL of the 10^−2^–10^−6^ dilutions were spotted onto the plates and incubated for 20 h at 37°C. Images in panels B-C are representative of at least three experiments giving similar results.

For western blot analysis of whole cell extracts, appropriate volumes of exponentially-growing bacterial cultures in MH in flasks were centrifuged, and pellets were suspended in SDS-PAGE loading buffer [0.25 M Tris-HCl pH 6.8, 2% (w/v) SDS, 10% (v/v) β-mercaptoethanol, 20% (v/v) glycerol]. Pellets from identical culture volumes were also collected to determine the cellular protein concentration for each sample by using the DC protein assay kit (Bio-Rad) and bovine serum albumin as the standard. Volumes of SDS-PAGE samples corresponding to 20 μg of proteins were loaded onto the gels. Proteins resolved by SDS-PAGE were electrotransferred onto a nitrocellulose filter (Hybond-C extra, Amersham) and probed for LptE or LptC using custom polyclonal rabbit antibodies and a goat anti-rabbit IgG HRP-conjugated secondary antibody (Sigma-Aldrich). Anti-LptE and anti-LptC antibodies were generated at GenScript (http://www.genscript.com/custom-polyclonal-antibody-production-services.html) using Keyhole Limpet Hemocyanin (KLH)-conjugated peptides as antigens (LptE epitope: RAAEPQQSPIEFPT; LptC epitope: NAHSLQYQEDGSLD). The epitopes were selected using the Optimum Antigen™ Design Tool (GenScript). Filters were developed with ECL chemiluminescent reagents (Amersham) and visualized in a ChemiDoc XRS+ system (Bio-Rad). When specified (Figure 1(b)), filters were developed with the Cyanagen Westar ηC Ultra 2.0 reagent and detected using an Odissey system (Li-Cor) system. To analyze the protein and LPS profiles across the sucrose gradient, aliquots from each fraction were subjected to immunoblot analysis. For the protein proﬁle of XcpY, 20 µL of each fraction were loaded on 12.5% SDS-PAGE gels and analyzed by immunoblotting using anti-XcpY antibodies [51]. To estimate the OprI and LPS distribution, 5 µL and 20 µL of each fraction, respectively, were separated by 18% Tricine-SDS-PAGE and immunoblotted using anti OprI antibodies [52] and anti-*P. aeruginosa* outer core specific antibodies (MEDIMABS). For western blot analysis of LptD-6His levels in LptE-depleted cells, 20 µL of isolated membranes were diluted in 5× SDS-PAGE loading buffer and proteins were separated by 10% SDS-PAGE followed by immunoblot analysis using anti-6His antibodies (Sigma-Aldrich), according to the manufacturer instruction. To analyze the oxidation state of LptD, equal amounts of membrane samples were treated with 5× SDS loading buffer with or without 5% (v/v) β-mercaptoethanol. Samples were boiled for 10 min before SDS-PAGE. For immunoblots with antibodies against XcpY, OprI, LPS and 6His, filters were visualized by immunofluorescence using IRDye secondary antibodies (Li-Cor) on an Odissey system (Li-Cor). Densitometric analysis of band intensities was performed using the Image Lab 3.0 software (Bio-Rad).

### *Galleria mellonella* infection assay

*P. aeruginosa* strains were grown in MH with 0.5% arabinose, and serial dilutions of bacterial cell suspensions in saline were injected into *G. mellonella* larvae as described [53]. Ten larvae were infected with each infecting dose in at least three independent experiments, and infected larvae were incubated at 30°C for up to 4 days to monitor mortality. Survival curves, LD_90_ and R^2^ values were determined using GraphPad Prism as previously described [54].

### Statistical analysis

Statistical analysis was performed with the software GraphPad Instat, using Kruskal-Wallis test followed by Dunn test for selected pairs of columns.

## Results

### LptE depletion only marginally affects *P. aeruginosa* growth

In order to investigate the role of LptE in LPS transport and cell viability in *P. aeruginosa*, we first attempted to generate a clean in-frame deletion in the *lptE* gene. However, several independent attempts failed, as the recombinant clones obtained after the insertion of the deletion mutagenesis plasmid into the chromosome always reverted to the wild type genotype during the second recombination event selected by the mutagenesis procedures (data not shown). We therefore decided to use a recently-developed strategy [44] to construct a conditional mutant carrying a copy of the *lptE* coding sequence in a neutral site of the genome (*attB*) under the control of an arabinose-dependent *araC*-P_BAD_ regulatory element and an in-frame deletion in the endogenous *lptE* gene. Surprisingly, we found that this conditional mutant was able to grow in MH in the absence of arabinose, although growth yields were slightly reduced as compared to those of the wild type PAO1 strain or the *lptE* conditional mutant grown in the presence of arabinose (Figure 1(a)). Growth of LptE-depleted cells was further reduced if these cells were subsequently refreshed in the absence of arabinose (second refresh in Figure 1(a)), while no additional growth defects were observed after further refreshes (data not shown). As *lptE* conditional mutant cells grown for two passages in the absence of the inducer showed a more severe growth deficiency as compared to cells directly refreshed from arabinose-containing medium (Figure 1(a)), also these cells (hereafter named *lptE* 2^nd^) were analyzed in subsequent assays. The effect of arabinose on the growth kinetics of the *lptE* conditional mutant showed a clear dose-dependent response (Fig. S2), indicating that the growth of this mutant is proportional to LptE expression levels. Western blot analysis with a polyclonal anti-LptE antibody revealed that LptE was strongly reduced in whole cell extracts of the *lptE* conditional mutant cultured under non-inducing conditions as compared to the wild-type strain (Fig. S3). Using a very sensitive detection system and appropriate sample dilutions, the LptE intracellular levels in the conditional mutant grown in the absence of arabinose were tentatively quantified as 8-fold lower (ca. 12.5%) than those of the wild type, while the amount of LptE further fell down to less than 6% of wild type levels in *lptE* mutant cells cultured for two subsequent passages in the absence of arabinose (*lptE* 2^nd^) (Figure 1(b)). It must be noted that LptE levels in the *lptE* conditional mutant cultured in the presence of 0.5% arabinose were only about 25–40% of the wild type levels (Figure 1(b) and S3), although growth of the mutant was restored to wild type rates (Figure 1(a) and S2). No further increase in LptE levels was observed with higher arabinose concentrations (up to 2%; data not shown). The partial restoration of LptE levels by arabinose can be explained by the fact that our conditional mutant is based on an *araC*-P_BAD_ regulatory element with a very weak ribosome-binding site (see Materials and Methods and ref. 44 for details). While this regulatory element is effective in lowering the basal, non-induced expression from *araC*-P_BAD_ in *P. aeruginosa* [36], it can also decrease its maximal induced activity, as recently demonstrated for similar constructs [55]. Interestingly, the amount of a cytoplasmic membrane protein involved in LPS transport (LptC) was comparable between LptE-replete and -depleted cells (Fig. S3), suggesting that LptE depletion does not negatively impact on the stability of the whole Lpt machinery. To investigate whether the residual growth of LptE-depleted cells is a general feature of the whole population or an adaptation occurring in a sub-population of cells, we determined the plating efficiency of the *lptE* conditional mutant in the presence or absence of arabinose. As shown in Figure 1(c), the number of colonies obtained for the *lptE* conditional mutant was comparable in MH agar plates with or without arabinose, although colonies were visually smaller in non-inducing conditions, confirming that also colony growth rates were slightly reduced for LptE-depleted cells. Similar results were obtained in a different complex medium (LB) and in the minimal medium M9 (Fig. S4). In addition, to rule out that the growth of the *lptE* conditional mutant under non-inducing conditions could be at least partly influenced by the appearance of sub-populations of fast-growing cells (carrying suppressor mutations), the strain was sequentially streaked on MH agar plates for several passages. Notably, no decrease in colony yields was observed over passages, nor did we detect colonies showing faster growth (Fig. S5).

### LptE depletion drastically impairs cell envelope integrity

**Figure 2.**
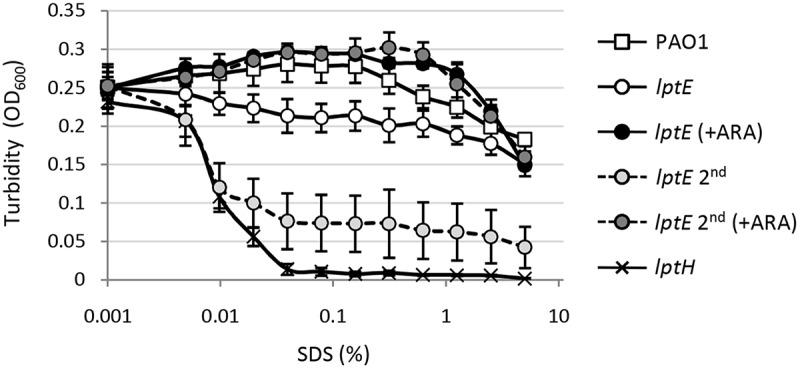
Effect of LptE depletion on cell envelope stability. Lytic effect of different SDS concentrations (0–5%), measured as decrease in cell suspension turbidity (OD_600_), on PAO1 and *lptE* conditional mutant cells grown in the presence and/or absence of 0.5% arabinose (ARA), corresponding to the first (*lptE*) or second refresh (*lptE* 2^nd^) described in the legend of Figure 1. As control, LptH-deficient cells (*lptH*), obtained through the culturing strategy described in Fig. S6, were included in the analysis. Values are the mean (±SD) of three independent experiments performed in duplicate.

**Figure 3.**
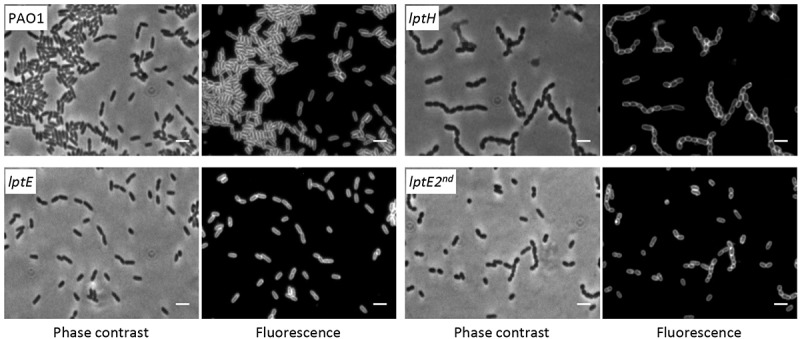
Phase contrast and fluorescence microscopy images of *P. aeruginosa* wild type PAO1, *lptH* and *lptE* conditional mutant cells grown in MH without arabinose, and stained with the membrane-binding dye FM^TM^ 5–95. *lptE* 2^nd^ corresponds to cells cultured for two subsequent passages in the absence of arabinose (see the legend of Figure 1 for details). Images are representative of several fields (≥10) showing comparable results. Scale bar: 3 µm.

LptE appears to be dispensable for *P. aeruginosa* growth and viability, nevertheless its depletion could impact on cell envelope integrity. In order to test this hypothesis, we determined the effect of LptE depletion on detergent and antibiotic resistance. Cell envelope integrity was first investigated through an SDS sensitivity assay [44]. Under non-permissive conditions, SDS resistance of the *lptE* conditional mutant was decreased as compared to the wild type, and was strongly defective in *lptE* cells cultured for two subsequent passages in the absence of arabinose (Figure 2). The SDS sensitivity profile of *lptE* cells at the second refresh under non-permissive conditions was almost comparable to that of LptH-depleted cells (Figure 2), which were collected using the culturing strategy described in Fig. S6, and used as control for a mutant with severe defects in envelope biogenesis due to the lack of an essential LPS transport protein [35]. Notably, arabinose completely restored SDS resistance in *lptE* conditional mutant cells to wild type levels (Figure 2). Microscopic analysis revealed that the morphology of LptE-depleted cells at the first refresh was overall comparable to that of wild type cells, while after two subsequent passages in the absence of arabinose LptE-depleted cells were slightly shorter and a small proportion of them appeared to grow as short cell chains (Figure 3). The chaining phenotype was much more severe in cells impaired in growth because of LptH depletion, which formed very long chains (Figure 3). This phenotype parallels that previously observed in *E. coli* mutants defective in LPS transport [12]. Both *lptH* and *lptE* conditional mutants showed a normal phenotype when cultured in the presence of arabinose (Fig. S7). To further investigate the contribution of LptE to the integrity of the OM barrier, the susceptibility of LptE-replete and -depleted cells to different antibiotics was investigated through the Kirby-Bauer disc diffusion assay (Table 1). As control we also tested antibiotic resistance of LptH-depleted cells, by culturing the *lptH* conditional mutant in the presence of the lowest concentration of arabinose, which supports residual growth (0.03%) [35]. Interestingly, the *lptE* conditional mutant grown without arabinose was much more sensitive to all antibiotics tested than the wild type strain, with the only exception of colistin (Table 1). In line with the SDS resistance profile (Figure 2), LptE-depleted cells cultured for two subsequent passages in the absence of arabinose showed further defects in resistance to many antibiotics, including colistin (Table 1). In spite of antibiotic-specific differences, the drug resistance profiles of LptE- and LptH-depleted cells were overall comparable (Table 1), suggesting that the OM barrier is strongly defective upon LptE depletion. Notably, addition of 0.5% arabinose to the growth medium only partially ameliorated the antibiotic susceptibility profile of the *lptE* conditional mutant, while it almost completely restored antibiotic resistance to wild type levels in the *lptH* conditional mutant (Table 1). Considering that our immunoblot assays showed that arabinose supplementation only partially re-establishes LptE levels in the *lptE* conditional mutant (Figure 1), it is worth hypothesizing that such reduced expression of LptE could account for the failure of arabinose to fully restore antibiotic resistance in the conditional mutant. To confirm this hypothesis, a plasmid for IPTG-inducible ectopic expression of LptE (pME*lptE*; Table S1) was introduced in the *lptE* conditional mutant. Immunoblot analysis confirmed that LptE levels in the *lptE* mutant harboring pME*lptE* were restored to wild type levels in the presence of 1 mM IPTG and 0.5% arabinose (Fig. S8). Notably, under these culturing conditions, susceptibility to most antibiotics was basically comparable between the *lptE* mutant harboring pME*lptE* and the wild type carrying the empty plasmid, although a slight decrease in resistance to streptomycin and rifampicin was still observed in the conditional mutant (Table 1). Overall, these assays clearly show that LptE-depleted cells are strongly defective in detergent and antibiotic resistance, which is indicative of major defects in cell envelope stability.

**Table 1.** Antibiotic susceptibility of *lpt* mutant strains by the Kirby-Bauer disk diffusion test^a.^

			Growth inhibition halo diameter (mm)^d^							
Strain^b^	ARA (%)^c^	IPTG (mM)^c^	Sm	Ery	Rif	Novo	Tob	Ipm	Cip	Ct
PAO1	0	0	12.4 (±1.0)	8.1 (±0.9)	7.1 (±0.2)	6.6 (±0.5)	22.3 (±0.5)	24.8 (±0.7)	31.1 (±0.7)	12.9 (±0.7)
*lptH*	0.03	0	18.7 (±2.3)*	22.3 (±2.1)**	33.3 (±3.1)***	14.3 (±0.6)*	31.7 (±2.1)**	39.7 (±2.5)**	44.7 (±2.3)***	18.0 (±1.0)*
	0.5	0	12.0 (±1.0)	7.3 (±0.6)	11.7 (±0.6)*	7.0 (±0.0)	23.3 (±1.5)	24.7 (±0.6)	27.7 (±0.6)	12.0 (±1.0)
*lptE*	0	0	21.3 (±1.7)**	17.1 (±2.4)**	18.5 (±1.7)**	17.0 (±1.4)**	26.2 (±1.6)*	28.7 (±1.9)*	37.3 (±2.0)**	12.9 (±0.7)
	0.5	0	20.2 (±1.0)*	13.9 (±1.1)*	18.0 (±0.8)**	16.0 (±0.5)*	23.7 (±1.5)	24.7 (±0.4)	35.8 (±1.5)*	12.5 (±0.6)
*lptE* 2^nd^	0	0	21.5 (±1.0)**	14.4 (±0.5)*	30.9 (±3.8)***	24.8 (±1.9)***	33.8 (±1.3)**	39.5 (±1.3)**	41.8 (±1.7)**	15.5 (±0.6)*
	0.5	0	19.8 (±0.5)*	14.5 (±0.6)*	15.8 (±1.0)*	16.3 (±1.5)*	25.5 (±0.6)	25.6 (±0.5)	35.1 (±1.3)*	12.9 (±0.3)
PAO1 pME6032	0	0	11.9 (±0.9)	7.6 (±0.5)	7.0 (±0.6)	7.3 (±0.5)	23.5 (±0.6)	25.8 (±1.3)	31.6 (±1.5)	12.5 (±0.6)
*lptE* pME6032	0	0	21.7 (±0.7)**	17.6 (±0.7)*	18.3 (±0.7)**	17.5 (±0.7)**	26.6 (±0.7)*	28.9 (±0.8)*	39.7 (±3.5)*	13.5 (±0.7)
*lptE* pME*lptE*	0	0	21.6 (±1.1)**	17.7 (±0.6)*	17.7 (±0.6)*	17.7 (±2.1)**	27.3 (±0.6)*	29.3 (±1.5)*	42.0 (±2.0)**	13.8 (±1.0)
	0.5	0	20.5 (±1.7)*	15.5 (±0.9)	18.3 (±1.3)**	17.0 (±1.4)**	24.5 (±1.7)	26.8 (±1.0)	39.3 (±1.0)*	13.2 (±0.4)
	0.5	1	15.5 (±1.3)	8.3 (±0.6)	10.5 (±0.6)	8.3 (±0.6)	23.7 (±1.5)	24.9 (±0.9)	34.4 (±1.8)	12.7 (±0.4)

^a^ Abbreviations: ARA, arabinose; Sm, streptomycin; Ery, erythromycin; Rif, rifampicin; Novo, novobiocin; Tob, tobramycin; Ipm, imipenem; Cip, ciprofloxacin; Ct, colistin.

^b^ The sample *lptE* 2^nd^ corresponds to the *lptE* conditional mutant pre-cultured in the absence of arabinose. All other conditional mutant samples were pre-cultured in the presence of 0.5% arabinose.

^c^ Arabinose and IPTG at the indicated concentrations had no effect on the antibiotic susceptibility profile of PAO1 and PAO1 pME6032, respectively (data not shown).

^d^ Values are the mean (± SD) of at least three independent assays. Asterisks and bold font indicate statistically-significant increases in antibiotic sensitivity with respect to PAO1 (or PAO1 pME6032): * *P *< 0.05, ** *P *< 0.01, *** *P *< 0.001 (Kruskal-Wallis).

## P. aeruginosa *LptE only moderately influences LPS transport*

**Figure 4.**
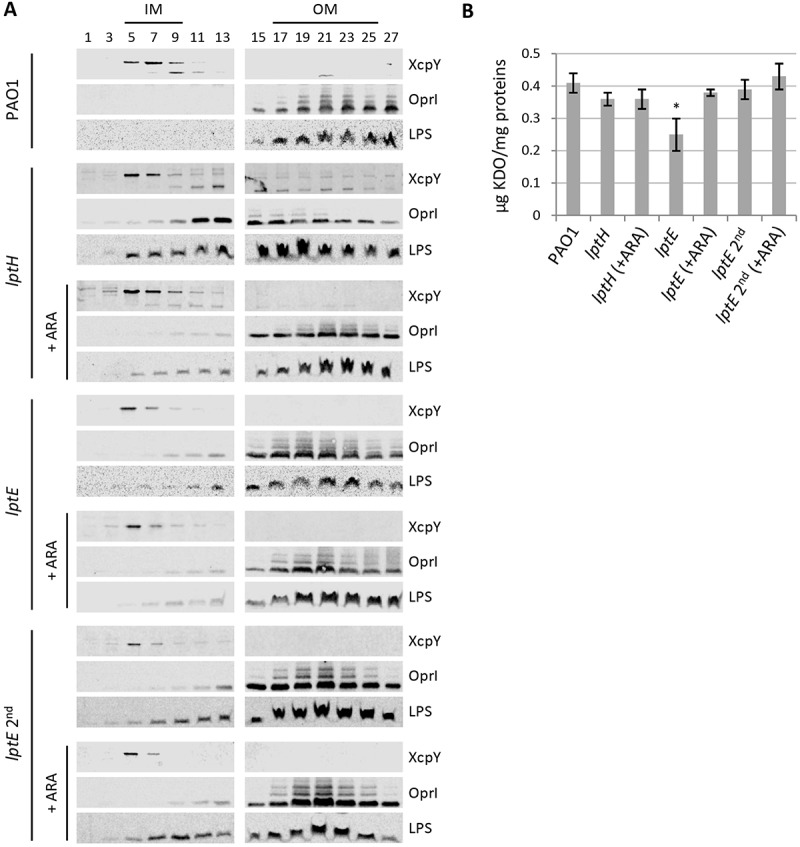
Localization and quantification of LPS. (a) Membrane fractionation of wild type PAO1, *lptH* and *lptE* conditional mutants. PAO1 and the *lptE* conditional mutant (*lptE*) were cultured in MH with or without 0.5% arabinose (ARA) as described in the legend to Figure 1. The sample *lptE* 2^nd^ corresponds to the *lptE* mutant pre-cultured in the absence of ARA (second refresh in Figure 1). PAO1 and *lptE* cells were collected for membrane fractionation when the cultures reached an OD_600_ = 0.8. As control, LptH-deficient cells (*lptH*), obtained through the culturing strategy described in Fig. S6, were included in the analysis. Membranes were fractionated by sucrose density gradient, and the different fractions were immunoblotted using antibodies against the IM protein XcpY, the OM protein OprI or LPS, as indicated on the right of each blot. Fractions corresponding to the IM or OM on the basis of the results obtained with the wild type PAO1 are indicated on the top of the figure. Images are representative of two experiments giving similar results. (b) Quantification of KDO in the above-mentioned strains cultured as described in panel A. Values are the mean (± SD) of three independent experiments. The asterisk indicates a statistically-significant decrease with respect to all other samples (*P *< 0.05, Kruskal-Wallis).

**Figure 5.**
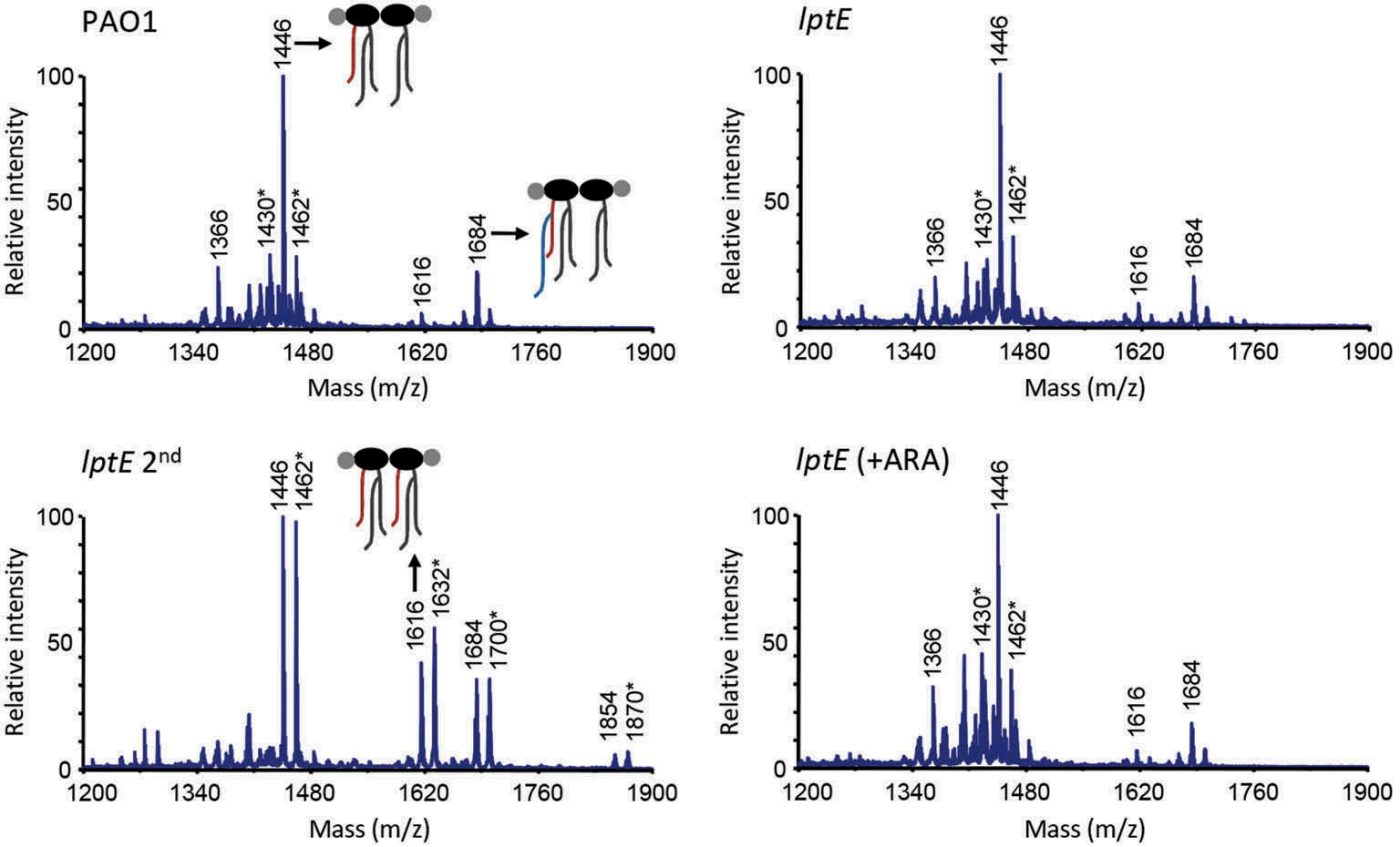
MALDI-TOF analysis of lipid A extracted from wild type (PAO1) and *lptE* mutant cells cultured in MH with or without 0.5% arabinose (ARA). *lptE* 2^nd^ corresponds to the *lptE* conditional mutant cultured for two subsequent passages in the absence of ARA (see the legend of Figure 1 for details). Spectra were obtained in the ion negative mode, thus m/z values correspond to (molecular mass – 1)/1. Spectra are representative of three biological replicates. Relevant lipid A forms are shown with cartoons: m/z = 1646, penta-acylated lipid A; m/z = 1616, hexa-acylated lipid A; m/z = 1684, penta-acylated lipid A with the addition of a palmitoyl group (in blue). Asterisks indicate lipid A forms that vary due to the removal (m/z −16) or addition (m/z + 16) of a hydroxyl group to the secondary C12 acyl chains. The chemical structures of the identified lipid A forms are shown in Fig. S9.

In order to verify whether the observed defects in cell envelope stability upon LptE depletion may correlate with impaired LPS transport, we performed membrane fractionation experiments to compare the amount and localization of LPS in LptE-replete and -depleted cells. We also used LptH-depleted cells as a positive control for the effect of an essential LPS transport protein on LPS localization [34,35]. Membranes were fractionated by sucrose density gradient centrifugation, as described in Materials and Methods, and the fractions were immunoassayed for LPS, as well as for XcpY and OprI as IM and OM markers, respectively [51,52]. As shown in Figure 4(a), in wild type cells the IM equilibrated around fractions 5–9 as judged by the distribution profile of XcpY, whereas the OM equilibrated around fractions 17–25 where the majority of both LPS and OprI fractionated. The LPS fractionation profile markedly changed in LptH-depleted cells, and was overall shifted towards lighter fractions as witnessed by the presence of LPS even in fractions where the IM XcpY protein equilibrates. Notably, the OprI fractionation profile paralleled that of LPS, indicating that the IM and OM could not be properly separated by buoyant-density centrifugation. A very small amount of LPS was shifted towards lighter fractions also in *lptH* mutant cells cultured under permissive conditions (0.5% arabinose), while no changes in the localization of the IM and OM markers XcpY and OprI were observed (Figure 4(a)). It thus appears that LPS transport is not fully restored to wild type levels in the *lptH* mutant grown in the presence of arabinose, albeit its antibiotic susceptibility profile was overall comparable to that of the PAO1 strain (Table 1). Collectively, these results indicate that LPS transport is severely impaired upon LptH depletion and are indicative of major defects in cell envelope organization, in line with the hypersensitivity of LptH-depleted cells to antibiotics and detergents (Figure 2 and Table 1) [35]. The fractionation profile of LptE-depleted cells was more similar to that of *lptH* mutant cells grown with arabinose. Indeed, some defects in LPS transport appeared in the *lptE* conditional mutant (*lptE* and *lptE* 2^nd^) as witnessed by appearance of small amount of LPS in lighter fractions. Nevertheless, LptE-replete and-depleted cells did not undergo the huge OM reorganization observed in LptH-depleted cells, since the fractionation profile of the IM and OM markers paralleled that observed in PAO1 (Figure 4(a)). Notably, the fractionation profile of the *lptE* conditional mutant was basically independent of the presence of arabinose in the culture medium, suggesting that the level of LptE does not impact on LPS localization. Although not quantitative, the immunoblots shown in Figure 4(a) suggest that LPS levels were somewhat reduced in LptE-depleted cells at the first refresh as compared to all other samples. We therefore quantified LPS levels by analyzing the amount of KDO, a specific component of LPS core oligosaccharide. The amount of KDO was overall comparable among wild type cells, LptH-depleted cells, and LptE-depleted cells at the second refresh, as well as all conditional mutants cultured in the presence of arabinose (Figure 4(b)). In contrast, a significant reduction (almost 40%) in KDO levels was observed in the *lptE* condition mutant at the first refresh without arabinose, in line with western blot results (Figure 4(a)). Finally, to confirm the correct localization of LPS in the outer leaflet of the OM, we analyzed the lipid A from wild type and *lptE* mutant cells by MALDI-TOF mass spectrometry (MS), to verify the presence of OM-specific lipid A modifications, *e.g*. PagL-mediated deacylation and PagP-mediated palmitoylation [56]. The MS spectra are shown in Figure 5, while the chemical structures of the identified lipid A forms are reported in Fig. S9. The lipid A profiles of wild type, LptE-replete mutant cells and LptE-depleted mutant cells (at the first refresh) were almost identical, with a major peak corresponding to mono-hydroxylated penta-acylated lipid A (m/z = 1446). This represents the main lipid A form in laboratory *P. aeruginosa* strains (e.g. PAO1 and PA14), and derives from PagL-mediated removal of the O-linked 3-OH-C10 acyl chain at position 3 of the hexa-acylated precursor (Fig. S9) [57,58]. Less abundant peaks have been identified as (i) different hydroxylation states of the penta-acylated lipid A (m/z = 1430 and 1462), (ii) penta-acylated lipid A palmitoylated by PagP (m/z = 1684), or (iii) the hexa-acylated lipid A precursor (m/z = 1616) (Figure 5 and S9). Some differences were instead observed in the lipid A profile of *lptE* mutant cells grown for two subsequent passages without arabinose (*lptE* 2^nd^). While the penta-acylated molecule remained the main lipid A form also in these cells, there was an increase in the hexa-acylated precursor and, to a minor extent, in the palmitoylated penta-acylated form (Figure 5). In addition, we also observed a relevant increase in the hydroxylation state of all these molecules (m/z = 1462, 1632 and 1700, respectively). Overall, this analysis strongly suggests that the majority of lipid A in LptE-depleted cells is localized in the outer leaflet of the OM, as ≥ 75% of peaks intensity corresponds to molecules modified by PagL or PagP (penta-acylated and palmitoylated lipid A), whose active sites face the external surface of the OM [56]. In conclusion, these data indicate that LptE is not directly involved in the transport and assembly of LPS into the OM and strongly suggest that lack of envelope integrity/stability in *lptE* mutant cells is not simply due to impaired LPS transport.

### LptE affects LptD levels

**Figure 6.**
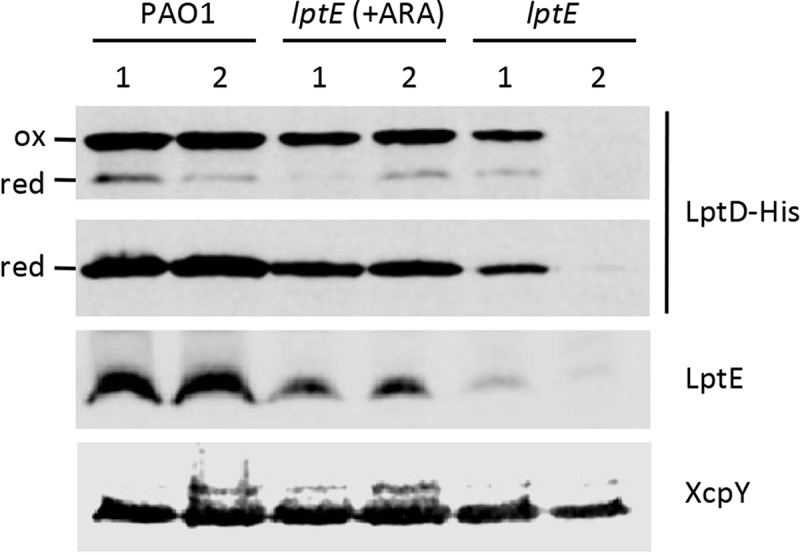
LptD levels in LptE-depleted cells. Total membrane samples were obtained from PAO1 *lptD-6his* and *lptE lptD-6his* cells grown as described in the legend of Figure 1. Cells were collected at OD_600_ = 0.8, both for the first (lanes 1) and the second refresh (lanes 2). Total membrane proteins corresponding to an OD_600_ = 1.6 were loaded in each lane. Filters were immunoblotted using antibodies raised against the 6His tag to detect LptD-6His, against LptE, or against XcpY, as indicated on the right of each blot. To analyze the oxidation state of LptD (red, reduced; ox, oxidized), membrane samples were treated with SDS loading buffer containing (lower panel) or not 5% β-mercaptoethanol (upper panel). Images are representative of three independent experiments giving similar results.

LptE directly participates to and is essential for LPS transport in *E. coli* [8], while it is dispensable for LPS transfer to the OM in *N. meningitidis* [24]. However, in both bacteria LptE was found to play an important role in the maturation of the integral OM component of the LPS transport machinery LptD, acting as a chaperone to stabilize LptD and/or assist LptD in folding and OM insertion [20–22,24]. We therefore sought to verify whether this chaperone function of LptE was also conserved in *P. aeruginosa*. To this aim, we first engineered the genome of the PAO1 and *lptE* conditional mutant strains to express a 6-His tagged LptD instead of the wild type protein (Table S1 and Fig. S1), and then compared the amounts of LptD-6His in membrane fractions from LptE-replete and -depleted cells by western blot analysis with an anti-6His antibody. LptD-6His levels were reduced in LptE-depleted cells as compared to wild type PAO1, and they fell to barely detectable levels in the *lptE* conditional mutant grown for two passages in the absence of arabinose (Figure 6). Growth of the *lptE* conditional mutant in the presence of arabinose partially restored LptD-6His levels, accounting for about 40% of the wild type levels (Figure 6). The majority of LptD was found in the oxidized (mature) form in all strains, with the only exception of *lptE* 2^nd^ samples, in which the amount of LptD was too low to draw conclusions about its oxidation state (Figure 6). Together, these results indicate that LptE is crucial for LptD maturation and/or stability also in *P. aeruginosa*, and suggest that, as previously observed in *N. meningitidis* [24], very low levels of LptD may be sufficient to allow insertion of LPS into the OM and, thus, to sustain growth of *P. aeruginosa*.

### LptE is important for *P. aeruginosa* virulence

**Figure 7.**
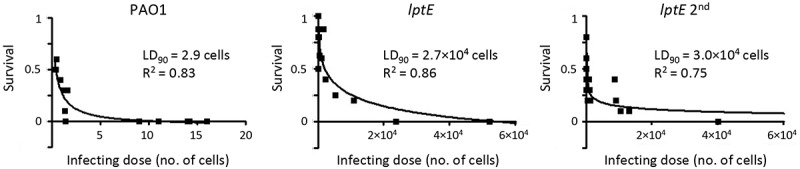
Effect of LptE depletion on *P. aeruginosa* virulence in *G. mellonella*. Survival curves of *G. mellonella* larvae infected with different doses of *P. aeruginosa* PAO1 or the *lptE* conditional mutant cultured in MH with 0.5% arabinose (PAO1 and *lptE*) or in MH without arabinose (*lptE* 2^nd^). Ten larvae were infected with each infecting dose in at least three independent experiments. LD_90_ and R^2^ values for each curve are shown.

The above results demonstrate that LptE-depleted cells of *P. aeruginosa* are able to grow under *in vitro* conditions (Figures 1 and S4) even if they appear to be strongly defective in cell envelope stability (Figure 2 and Table 1). To evaluate the impact of LptE depletion on *P. aeruginosa* virulence, we tested the *lptE* conditional mutant in a simple model of acute infection based on the larvae of the insect *G. mellonella* [53]. This infection model was successfully used by our group to verify and compare the pathogenicity of *P. aeruginosa* arabinose-dependent conditional mutants in different essential genes [35,44,59]. *G. mellonella* larvae were infected with different doses of either the wild type or the *lptE* conditional mutant, pre-cultured either in MH with 0.5% arabinose or in the absence of arabinose (*lptE* 2^nd^ in Figure 7), and lethality was monitored for 4 days. The LD_90_ (*i.e*. the number of bacterial cells that is theoretically required to kill 90% of the larvae) inferred from survival curves was about 3 cells for the wild type PAO1, in line with previous reports [35,44,59], while it was about 9,000-and 10,000-fold higher for the *lptE* conditional mutant pre-cultured with or without arabinose, respectively (Figure 7). This infection assay demonstrates that LptE depletion severely impairs the pathogenicity of *P. aeruginosa* in *G. mellonella* larvae, suggesting that this protein can play a relevant role during the *P. aeruginosa* infectious process. Notably, the defect in infectivity of LptE-depleted cells is however not comparable to that of a mutant completely impaired in LPS transport, *i.e*. the *lptH* conditional mutant, which showed an LD_90_ of 1.6 × 10^7^ cells in the same animal model [35], more than 500-fold higher than that of the *lptE* conditional mutant (Figure 7).

## Discussion

In the last decade, several research groups have made significant efforts to understand the molecular and biochemical bases of LPS transport across the cell envelope and insertion into the OM. However, the majority of the information gained on this crucial OM biogenesis process derives from studies involving only two model Gram-negative bacteria, *E. coli* and the non-LPS-dependent *N. meningitidis* (reviewed in [6,60–64]). Notably, detailed investigation of LPS transport in only two organisms was sufficient to identify a major distinctive feature of the Lpt machinery. Indeed, while in *E. coli* the Lpt machinery consists of seven essential components, the *N. meningitidis* Lpt machinery can work as a six-component complex, since *lptE* gene deletion does not impair LPS transport to the cell surface [24]. In this work, we sought to verify the role of LptE in LPS transport in *P. aeruginosa* through conditional mutagenesis. In our *P. aeruginosa lptE* conditional mutant, we found that LptE depletion only marginally affects LPS localization and maturation (Figures 4 and 5). Moreover, arabinose-mediated increase in LptE expression does not change the LPS profile of the *lptE* conditional mutant (Figure 4), indicating that LPS localization is not strictly dependent on LptE levels. These results suggest that LptE could not be directly involved in LPS transport in *P. aeruginosa*, similarly to what observed in *N. meningitidis* [24] but in contrast with its proposed LPS-binding role in *E. coli* [8,20,25]. This hypothesis would be indirectly supported by a recent report showing that *P. aeruginosa* LptE has very weak affinity for LPS *in vitro* [65]. On the other hand, as in *E. coli* and *N. meningitidis* [20–22,24], LptE appears to be crucial for LptD biogenesis and/or stability also in *P. aeruginosa* (Figure 6), indicating that the chaperone function of LptE may be conserved in Gram-negative bacteria. It is interesting to note that both bacterial growth and LPS transport were only slightly impaired in *P. aeruginosa* LptE-depleted cells in spite of the low levels of LptD (Figures 1 and 4), as previously reported also for the *N. meningitidis lptE* mutant [24]. Considering that the essentiality of LptD in *P. aeruginosa* has been experimentally demonstrated [33], this evidence suggests that strongly reduced amounts of LptD (and LptE) may be sufficient to sustain LPS transport and growth in *P. aeruginosa*, as previously observed in *N. meningitidis* [24]. Notably, LptE-depleted cells were proficient in growth under *in vitro* conditions, but strongly defective in detergent and antibiotics resistance (Figure 2 and Table 1). This is suggestive of major defects in cell envelope biogenesis and/or integrity, which could reasonably account for the significantly reduced virulence of LptE-depleted cells in the *G. mellonella* model of infection (Figure 7). Notably, no relevant differences were observed in the infectivity of LptE-depleted cells pre-cultured or not with arabinose (Figure 7), even if these cells displayed different susceptibility levels to SDS and antibiotics (Figure 2 and Table 1). Although we are aware that is not easy to directly correlate results from *in vitro* assays with the outcome of infection models, this finding suggests that either maximum LptE depletion is reached earlier during *in vivo* growth as compared to *in vitro* conditions or, alternatively, that the strongly reduced infectivity of LptE-depleted cells might (also) be due to some additional role(s) of LptE during infection other than its relevant effect on cell envelope stability. It is also interesting to note that *P. aeruginosa* sensitivity to detergents and antibiotics showed differential dependence on LptE and LptD levels. Indeed, partial complementation of LptE and LptD expression in the *lptE* conditional mutant grown in the presence of arabinose (Figures 1, S3 and 6) was sufficient to fully re-establish wild type levels of SDS resistance (Figure 2), but only moderately restored antibiotics resistance (Table 1). Antibiotics resistance in the *lptE* conditional mutant was however re-established at almost wild type levels upon complete restoration of LptE levels through plasmid-mediated expression (Table 1). Moreover, the LPS profile of the *lptE* conditional mutant was very similar to that of the *lptH* conditional mutant grown in the presence of 0.5% arabinose (Figure 4), whereas only the former was strongly defective in drug resistance (Table 1), implying that the high antibiotic sensitivity of the *lptE* mutant reasonably does not depend on its slightly impaired LPS transport. Overall, our data suggest that the detrimental effect on cell envelope integrity caused by LptE depletion could involve two different mechanism(s). First, reduced LptE expression strongly impairs LptD maturation, and although *P. aeruginosa* seems to tolerate low LptD levels, LptD depletion likely causes slight defects in LPS transport (Figure 4) and, consequently, in cell division (Figure 3), that could represent the basis for the high SDS sensitivity of LptE-depleted cells, as previously observed for other *P. aeruginosa* mutants defective in cell division [44]. Second, LptE depletion may result in a fraction of LptD channels in the OM that are not properly stabilized and plugged by LptE. Improperly folded and/or un-plugged LptD channels in the OM could compromise the OM barrier, ultimately leading to impaired antibiotic resistance, as previously reported in *E. coli* [66]. This would also be in agreement with a recent work showing that the replacement of the *P. aeruginosa lptD* gene with defective alleles, encoding LptD variants with a deletion in a loop predicted to interact with the LptE plug, drastically increases the susceptibility of *P. aeruginosa* to several classes of antibiotics [67]. Recently, the structure of the *P. aeruginosa* LptD/E has been solved and compared to that of other Gram-negative bacteria [68]. LptD/E structures exhibit an identical fold, although the *P. aeruginosa* LptD/E complex slightly differs due to (i) a larger luminal volume, composed of two cavities in contrast to only one in other structures, and (ii) the presence of additional insertion loops in the extracellular region of the barrel, conferring a larger molecular surface [68]. Moreover, *P. aeruginosa* LptD carries a long (~ 90-aa) insertion at the N-terminus, which is absent in other LptD homologues [34,68]. The biological implication of these unique properties of *P. aeruginosa* LptD/E is not clear. While it has been proposed that they could be due to the specific structure of the *P. aeruginosa* LPS [64], it cannot be ruled out that such structural features could (also) account for a specific maturation process and/or additional functions of the *P. aeruginosa* LptD/E complex. An aspect that deserves further investigation is the effect of LptE depletion on LPS biogenesis and maturation. Our data suggest that reduced LptE expression can cause a reduction in LPS biogenesis, while LPS transport and maturation are not or only slightly affected (Figures 4 and 5). Notably, after prolonged LptE depletion (*lptE* 2^nd^ samples), *P. aeruginosa* cells appear to restore normal LPS biosynthesis levels, although the hydroxylation state of lipid A is considerably increased (Figures 4 and 5). *P. aeruginosa* has two homologues (LpxO1 and LpxO2) of the lipid A-specific hydroxylase LpxO first characterized in *Salmonella*, which are likely responsible for the hydroxylation of the two secondary acyl chains of lipid A in *P. aeruginosa* (Fig. S9) [58]. Although the specific role of lipid A hydroxylation in *P. aeruginosa* has not been directly investigated yet, it has been hypothesized that changes in the hydroxylation state of lipid A could influence the fluidity and/or permeability of the OM [69,70]. At present, however, we cannot state whether the observed increase in lipid A hydroxylation represents a specific strategy of *P. aeruginosa* to adapt to an LptE/LptD-depleted LPS transport machinery or, alternatively, a general stress response of cells growing with slightly impaired LPS insertion into the OM. Studies involving the generation of *lpxO1/2* single and double mutants are clearly required to elucidate the function and/or effect of lipid A hydroxylation in *P. aeruginosa*. Finally, it should be noted that, while LptE depletion strongly impaired LptD folding and stability (Figure 6), in our study we did not observe the appearance of any aminorabinosylated lipid A forms (Figure. 5 and S9), which were instead found to accumulate in a *P. aeruginosa lptD* conditional mutant cultured with a very low inducer concentration [33]. Whether these differences in lipid A modifications are due to experimental/technical issues (e.g. different culture media, different growth rates) or to different adaptive responses to LptE or LptD depletion remains to be determined. In conclusion, this work provides evidence that LptE plays a crucial role in *P. aeruginosa* due to its importance as chaperone and plug for LptD. It should be noted that, while our *lptE* conditional mutant is viable and grows *in vitro* with strongly reduced LptE levels, many attempts to obtain a clean *lptE* deletion mutant were unsuccessful (see Results). Therefore, it is plausible that the growth phenotype of the *lptE* conditional mutant is influenced by such residual, low-level expression of LptE, making it impossible to definitely conclude whether LptE represents a *bona fide* essential protein in *P. aeruginosa*. To date, the role of LptE in LPS transport and cell physiology has only been investigated in *E. coli, N. meningitidis* and *P. aeruginosa*, although bioinformatics analyses have identified LptE homologues in almost all *Proteobacteria* and in some non-proteobacterial LPS-producing Gram-negatives [24,25,60]. It would therefore be interesting to investigate to what extent the roles of LptE in LPS transport, LptD maturation and/or its LPS transport-independent effect on cell envelope homeostasis are conserved among Gram-negative bacteria. This information could also have important clinical implications, also considering that the LptD/E complex has been pharmacologically validated as a promising antibiotic target *in vitro* [32,33]. Understanding how the LptD/E translocon works in different bacteria and the effect of its depletion on bacterial pathogenicity, *in vivo* fitness and resistance could indeed drive the development of further compounds targeting this crucial cell envelope biogenesis step with narrow- or broad-spectrum activity against Gram-negative pathogens.
