# Supplementary material for: Pseudomonas aeruginosa LptE is crucial for LptD assembly, cell envelope integrity, antibiotic resistance and virulence
Source: Virulence. 2018 Nov 4;9(1):1718–33. doi: 10.1080/21505594.2018.1537730 (PMC7204523; doi:10.1080/21505594.2018.1537730)
Supplement: Supplemental Material [file kvir-09-01-1537730-s0001.pdf]

***Pseudomonas aeruginosa* LptE is not directly involved in lipopolysaccharide transport but is crucial for LptD assembly, cell envelope integrity, antibiotic resistance and infectivity**

Alessandra Lo Sciuto[a*], Alessandra M. Martorana[b*], Regina Fernández-Piñar[a], Carmine Mancone[c], Alessandra Polissi[b], and Francesco Imperi[a]#

Table S1. Bacterial strains and plasmids used in this study

Strain or plasmid	Genotype and/or relevant characteristics	Reference or source
P. aeruginosa		
PAO1 (ATCC15692)	Prototroph	American type culture collection
lptE	*lptE* conditional mutant of PAO1, deleted of the *lptE* coding sequence and carrying *a*n arabinose-dependent copy of *lptE*	This work
lptH	*lptH* conditional mutant of PAO1, deleted of the *lptH coding* sequence and carrying an arabinose-dependent copy of *lptH*	Fernandez-Pinar *et al.*, 2015
PAO1 *lptD-6his*	PAO1 carrying a 6his-tag sequence fused in frame to the 3' end of the *lptD* gene	This work
lptE lptD-6his	*lptE* conditional mutant carrying a 6his-tag sequence fused in frame to the 3' end of the *lptD* gene	This work
E. coli		
S17.1*λpir*	*thi pro hsdRhsdM$^+$recA RP4-2-Tc::Mu-Km::Tn7 λpir,* Sm^R	Simon *et al.*, 1983
**Plasmid**		
pBluescript II KS+ (pBS)	Cloning vector; ColE1 replicon; Ap^R	Stratagene
pDM4	Suicide vector; *sacB*^{R}, *oriR6K*; Cm^R	Milton *et al.*, 1994
pDM4Δ*lptE*	pDM4 derivative for *lptE* in-frame deletion	This work
mini-CTX1-*araC*P_{BAD}*tolB*	mini-CTX1 derivative carrying the *araC*P_{BAD}*tolB*, Tc^R	Lo Sciuto *et al.*, 2014
mini-CTX1-*araC*P_{BAD}*lptE*	mini-CTX1-*araC*P_{BAD}*tolB* derivative in which *tolB* has been replaced with the *lptE* coding sequence	This work
pME6032	IPTG inducible expression vector, Tc^R	Heeb *et al.*, 2002
pME*lptE*	pME6032-derivative carrying an IPTG-inducible copy of *lptE*	This work
pDM4*lptD-6his*	pDM4 derivative for replacement of *lptD* with *lptD-6his*, encoding an LptD protein with a 6-His tag at the C-terminus	This work

References

Heeb S, Blumer C, Haas D. Regulatory RNA as mediator in GacA/RsmA-dependent global control of exoproduct formation in *Pseudomonas fluorescens* CHA0. J Bacteriol. 2002;184:1046-1056.

Fernández-Piñar R, Lo Sciuto A, Rossi A, et al. *In vitro* and *in vivo* screening for novel essential cell-envelope proteins in *Pseudomonas aeruginosa*. Sci Rep. 2015;5:17593.

King JD, Kocíncová D, Westman EL, Lam JS. Review: Lipopolysaccharide biosynthesis in *Pseudomonas aeruginosa*. Innate Immun. 2009;15:261-312.

Lo Sciuto A, Fernández-Piñar R, Bertuccini L, et al. The periplasmic protein TolB as a potential drug target in *Pseudomonas aeruginosa*. PLoS One. 2014;9:e103784.

Milton DL, O'Toole R, Horstedt P, Wolf-Watz H. Flagellin A is essential for the virulence of *Vibrio anguillarum*. J Bacteriol. 1996;178:1310-1319.

Simon R, Priefer U, Pühler A. A broad host range mobilization system for *in vivo* genetic engineering: transposon mutagenesis in Gram negative bacteria. Bio/Technology. 1983;1:784-790.

Table S2. Primers used in this study [a]

**Primer name**	**Sequence (5'→3')**[b]	**Restriction site**	**Application**
*lptE*_FW	GGAGTGAGGGAAGCTTGATG	HindIII	Generation of mini-CTX1-*araC*P_{BAD}*lptE*
*lptE*_RV	cggaattcTCACGGGGTGGGGAACTC	EcoRI	Generation of mini-CTX1-*araC*P_{BAD}*lptE*
lptE mut_UP_FW	ccgctcgAGGCCAGCCAGGATGTCG	XhoI	Generation of pDM4Δ*lptE*
lptE mut_UP_RV	cgggatcCGGCGCTGGTCAGGATAC	BamHI	Generation of pDM4Δ*lptE*
lptE mut_DOWN_FW	cgggatccTGAAGGTCACCAGCAACGC	BamHI	Generation of pDM4Δ*lptE*
lptE mut_DOWN_RV	gctctagaTCACGGGGTGGGGAACTC	XbaI	Generation of pDM4Δ*lptE*
*lptE*_pME6032_FW	cggaattCCGGGGGCGCTGATCAAC	EcoRI	Generation of pME*lptE*
*lptE*_pME6032_RV	ccgctcgagTCACGGGGTGGGGAACTC	XhoI	Generation of pME*lptE*
*lptD-6his*_UP_FW	gctctaGACTGGCGCATCAACTCCG	XbaI	Generation of pDM4*lptD-6his*
*lptD-6his*_UP_RV	cggaattcaatggtgatggtgatggtgCATAGCTTG ATCTTCACGTTG	EcoRI	Generation of pDM4*lptD-6his*
*lptD-6his*_DOWN_FW	cggaattCAAGACGGAAATGTTCCTCG	EcoRI	Generation of pDM4*lptD-6his*
*lptD-6his*_DOWN_RV	ccgctcgAGTTCTTCACCTCCTGCTCG	XhoI	Generation of pDM4*lptD-6his*
*6his*_check_FW	CACCATCACCATCACCATTGA		PCR screening of *lptD-6his* recombinant strains
M13FW	GTTTTCCCAGTCACGAC		Sequencing from pBS
M13RV	CAGGAAACAGCTATGAC		Sequencing from pBS
P_{BAD}_FW	CATAAGATTAGCGGATCCTAC		Sequencing from mini-CTX1-*araC*P_{BAD} constructs

[a] Preparative PCRs for cloning were performed using the genomic DNA of *P. aeruginosa* PAO1 as the template.
[b] The restriction site used for cloning is underlined in the primer sequence.

A

GACTGGCGCATCAACTCCGACTTCAATTGGAACCCCAACACCAGTCGTACTGAGTCCGGTAGCGCCATCTTCCACTACCA
GCCCGAAGTCGATCCGGGCAAGGTGGTCAACGTTGGCTATCGCTATCGTGCAGATGCTCGGCGCTTTGATTCCTCCCGCG
GCACATTCCGATATGGCAACGAAAACGACATCATCAAGCAGCACGATTTCTCGGTCATCTGGCCGCTGGTGCCGCAGTGG
TCCGTGCTCGCCCGCTGGCAGTACGACTACAACAAGAACCGCACCCTCGAGGCCTTCGGCGGCTTCGAATACGACAGCTG
CTGCTGGAAGCTGCGCCTGATCAACCGCTACTGGCTGGATGTGGACGATGACGCCTTCCTCGTGCAGAGCGAGAAGGCCG
ACCGCGGCATCTTCCTGCAGATCGTCCTGAAGGGCCTCGGCGGCATCGTAGGCAACAAGACGGAAATGTTCCTCGACAAA
GGCATCCAAGGTTATCGTCAACGTGAAGATCAAGCTATGTAACCGGCTGCGCCCCCTTGCATTGGGCGCAGCACTCCTTT
GCTCCTTCGCGCATGCCGAAGTGGTACCACTGGACCGGGTCGTCGCCATCGTCGACAACGATGTGATCATGCAGAGCCAG
CTCGACCAGCGCCTGCGTGAAGTGCACCAGACGCTGCTCAAGCGCGGTGCGCCGCTGCCGCCCGAGCACGTGCTGACCCA
GCAGGTGCTGGAACGCCTGATCATCGAGAACATCCAGCAGCAGATCGGCGACCGCTCCGGCATCCGCATCTCCGACGAGG
AGCTGAACCAGGCGATGGGCACCATCGCCCAGCGCAACGGCATGAGCCTGGAGCAGTTCCAGACCGCCCTCACCCGCGAC
GGCTTGTCCTACGCCGACGCCCGCGAGCAGGTCCGTCGCGAGATGGTGATCAGCCGTGTGCGCCAGCGCCGGGTCGCCGA
GCGCATCCAGGTCAGCGAGCAGGAGGTGAAGAACT

B

ACACCAGTCGTACTGAGTCCGGTAGCGCCATCTTCCACTACCAGCCCGAAGTCGATCCGGGCAAGGTGGTCAACGTTGGC
TATCGCTATCGTGCAGATGCTCGGCGCTTTGATTCCTCCCGCGGCACATTCCGATATGGCAACGAAAACGACATCATCAA
GCAGCACGATTTCTCGGTCATCTGGCCGCTGGTGCCGCAGTGGTCCGTGCTCGCCCGCTGGCAGTACGACTACAACAAGA
ACCGCACCCTCGAGGCCTTCGGCGGCTTCGAATACGACAGCTGCTGCTGGAAGCTGCGCCTGATCAACCGCTACTGGCTG
GATGTGGACGATGACGCCTTCCTCGTGCAGAGCGAGAAGGCCGACCGCGGCATCTTCCTGCAGATCGTCCTGAAGGGCCT
CGGCGGCATCGTAGGCAACAAGACGGAAATGTTCCTCGACAAAGGCATCCAAGGTTATCGTCAACGTGAAGATCAAGCTA
TGCACCATCACCATCACCATTGAATTCAAGACGGAAATGTTCCTCGACAAAGGCATCCAAGGTTATCGTCAACGTGAAGA
TCAAGCTATGTAACCGGCTGCGCCCCCTTGCATTGGGCGCAGCACTCCTTTGCTCCTTCGCGCATGCCGAAGTGGTACCA
CTGGACCGGGTCGTCGCCATCGTCGACAACGATGTGATCATGCAGAGCCAGCTCGACCAGCGCCTGCGTGAAGTGCACCA
GACGCTGCTCAAGCGCGGTGCGCCGCTGCCGCCCGAGCACGTGCTGACCCAGCAGGTGCTGGAACGCCTGATCATCGAGA
ACATCCAGCAGCAGATCGGCGACCGCTCCGGCATCCGCATCTCCGACGAGGAGCTGAACCAGGCGATGGGCACCATCGCC
CAGCGCAACGGCATGAGCCTGGAGCAGTTCCAGACCGCCCTCACCCGCGACGGCTTGTCCTACGCCGACGCCCGCGAGCA
GGTCCGTCGCGAGATGGTGATCAGCCGTGTGCGCCAGCGCCGGGTCGCCGAGCGCA

Figure S1. Genomic region encompassing the 3’ end of *lptD* and the 5’ end of *surA* in (A) the wild type strain *P. aeruginosa* PAO1 (www.pseudomonas.com), and (B) the recombinant strains PAO1 *lptD-6his* and *lptE lptD-6his* (Table S1). The DNA region in panel B was verified by DNA sequencing for both PAO1 *lptD-6his* and *lptE lptD-6his*. The stop codon of *lptD* and the start codon of *surA* are highlighted in red and green, respectively. The annealing region of primers used for pDM4*lptD-6his* construction are highlighted in grey in panel A (from top to bottom: *lptD6his*_UP_FW, *lptD6his*_DOWN_FW, *lptD6his*_UP_RV, *lptD6his*_DOWN_RV; Table S2). The 6His-tag encoding sequence and the DNA region duplicated in the PAO1 *lptD-6his* and *lptE lptD-6his* genomes are highlighted in yellow and underlined, respectively (panel B).

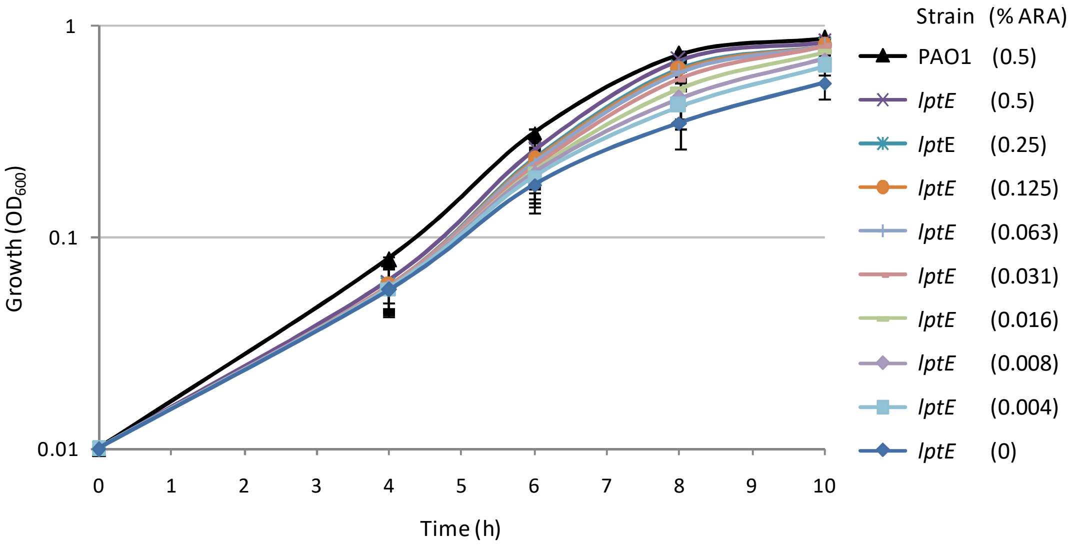

Figure S2. Growth of *P. aeruginosa* PAO1 and the *lptE* conditional mutant in 96-well microtiter plates in MH broth supplemented with increasing concentrations of arabinose (ARA, 0-0.5%) at 37°C and vigorous shaking (200 rpm). Results are the mean (± SD) of three independent experiments performed in triplicate.

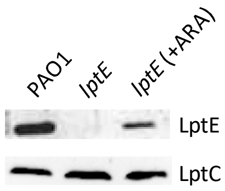

Figure S3. Intracellular levels of LptE and LptC in PAO1 and *lptE* cells grown for 14 h in MH supplemented or not with ARA, determined by western blot analysis of whole cell lysates (20 µg of proteins) with antibodies against LptE or LptC, as indicated on the right of each blot. Filters were developed with ECL chemiluminescent reagents and visualized in a ChemiDoc XRS+ system.

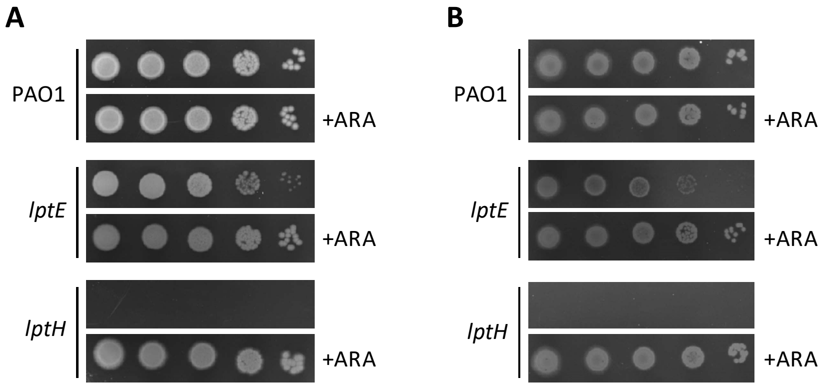

Figure S4. Colony growth of *P. aeruginosa* PAO1, the *lptE* conditional mutant and the *lptH* conditional mutant (used as control) on LB (panel A) or M9 minimal medium supplemented with 20 mM succinate and 50 μM $FeCl_3$ (panel B), in the presence or in the absence of 0.5% arabinose (ARA). Exponential phase cultures in LB or M9 with 0.5% ARA were normalized to OD_{600} = 1 in saline, and 5 μl of the 10^{-2}-10^{-6} dilutions were spotted onto the plates and incubated for 20 h at 37°C. The images are representative of two independent experiments giving similar results.

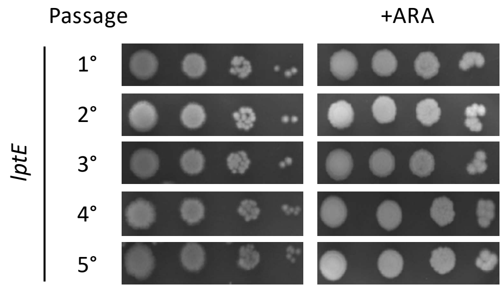

Figure S5. Colony growth of the *P. aeruginosa lptE* conditional mutant cultured for up to 5 subsequent passages on MH agar plates. At each passage, several colonies from MH agar without arabinose (ARA) were resuspended in saline, normalized to OD_{600} = 1 in saline, and 5 μl of the 10^{-3}-10^{-6} dilutions were spotted onto MH agar with or without 0.5% ARA. Pictures were taken after 20 h incubation at 37°C. The images are representative of two independent experiments giving comparable results.

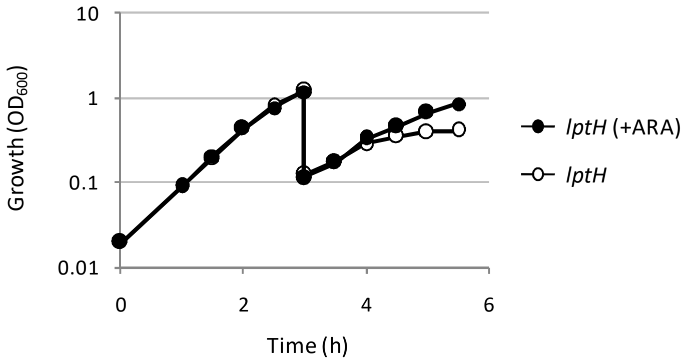

Figure S6. Growth of the *lptH* conditional mutant at 37°C in MH at 200 rpm in flasks after two successive subcultures in the presence (filled symbols) or in the absence (open symbols) of 0.5% arabinose (ARA), in order to obtain cells depleted of LptH. Briefly, bacteria were cultured for 14 h in MH supplemented with 0.5% ARA (not shown in the figure) and then diluted 1:100 in fresh medium with or without ARA (time 0). After 3 h of growth, cultures were diluted again 1:10 in fresh medium and incubated at 37 °C. The graph is representative of several assays giving similar results.

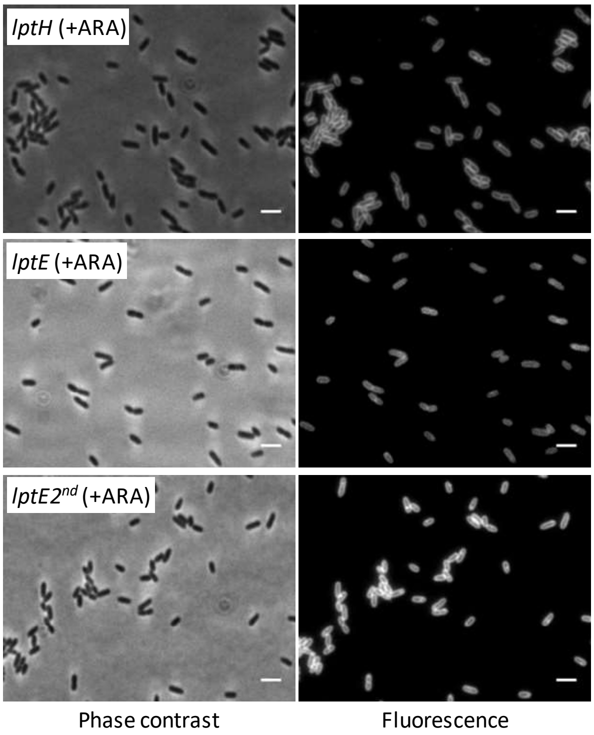

Figure S7. Phase contrast and fluorescence microscopy images of *P. aeruginosa lptH* and *lptE* conditional mutant cells grown in the presence of 0.5% arabinose and stained with the membrane-binding dye FM^{TM} 5-95. Images are representative of several fields (≥ 10) showing comparable results. Scale bar: 3 µm.

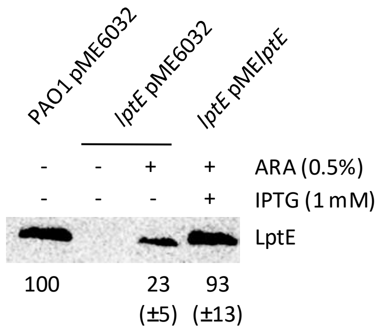

Figure S8. Intracellular LptE levels in PAO1 pME6032, *lptE* pME6032 and *lptE* pME*lptE* cells grown for 14 h in MH supplemented or not with 0.5% ARA and 1 mM IPTG, determined by Western blot analysis of whole cell lysates (20 µg of proteins) using the anti-LptE antibody. The image is representative of three assays giving comparable results. Quantification of LptE levels is reported below each lane, expressed as percentage (mean±SD) of the amount present in the wild type carrying the empty plasmid.

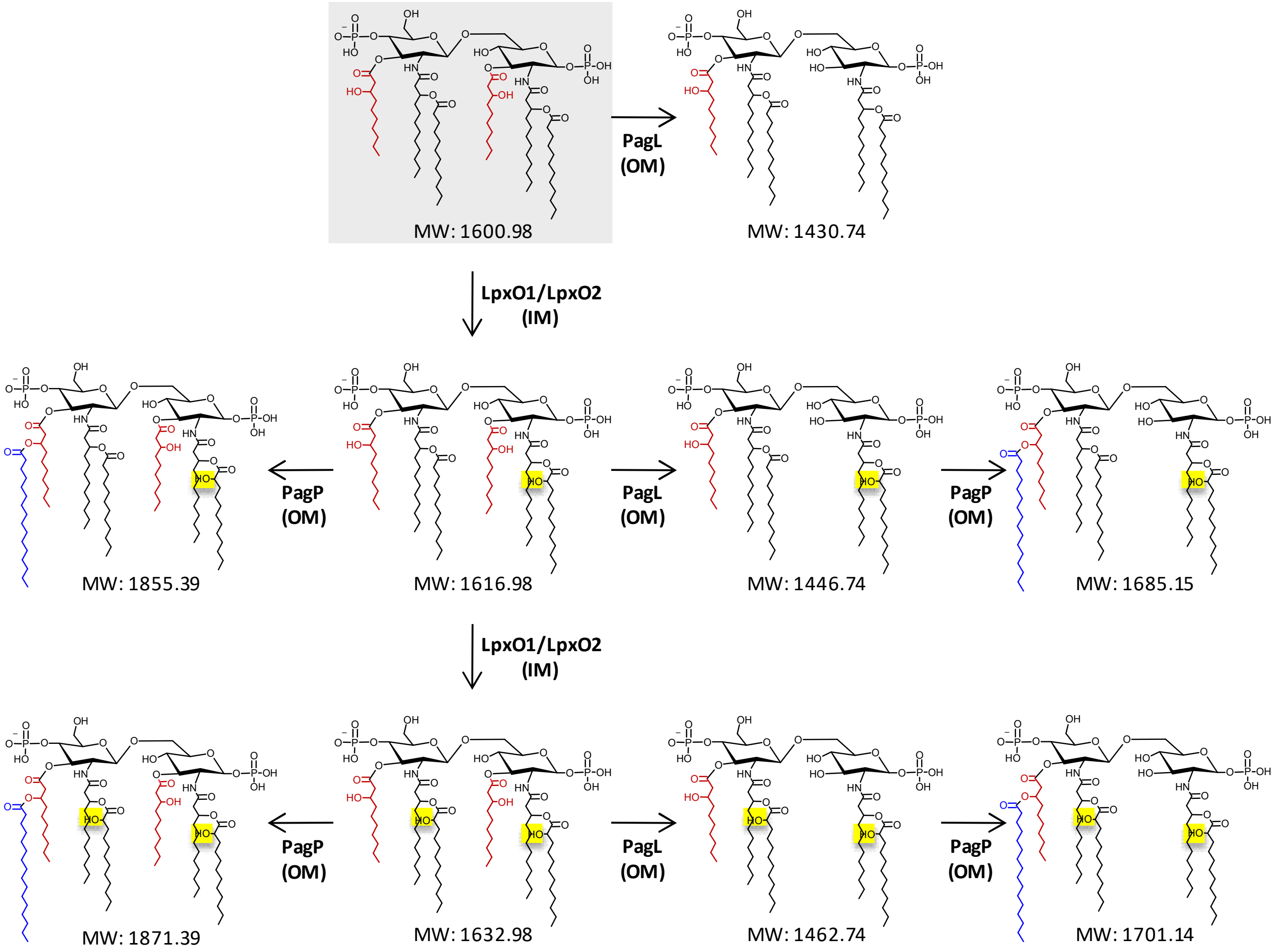

Figure S9. Chemical structure and predicted molecular weight (MW) of the main lipid A forms identified in *P. aeruginosa* PAO1 and/or in the *lptE* conditional mutant. 3-OH-C10 and C16 (palmitoyl) acyl chains are shown in red and blue, respectively. The molecule highlighted in grey has not been observed in our MS spectra (Fig. 5), but represents the unmodified lipid A precursor synthesized by *P. aeruginosa* (King *et al.*, 2009). The enzymes responsible for lipid A modifications are shown, together with their subcellular localization (IM, inner membrane; OM, outer membrane). Chemical structures were drawn with the ACD/ChemSketch software, which also calculated their MW. The hydroxyl groups predicted to be attached by LpxO1 and/or LpxO2 are highlighted in yellow.
